# Maternal Risk Factors Triggering Congenital Heart Defects in Down Syndrome: A Case-Control Study

**DOI:** 10.3390/pediatric14010015

**Published:** 2022-02-28

**Authors:** Ambreen Asim, Sarita Agarwal, Deepika Delsa Dean

**Affiliations:** Department of Medical Genetics, Sanjay Gandhi Postgraduate Institute of Medical Sciences (SGPGIMS), Lucknow 226014, India; ambreen13021988@gmail.com (S.A.); deepikadean.ddd@gmail.com (D.D.D.)

**Keywords:** Down syndrome, congenital heart defects, MTHFR, MTRR

## Abstract

Objectives: Maternal MTHFR and MTRR polymorphisms as a risk of CHD in DS fetus were studied along with maternal folic acid supplementation, which could influence the folate metabolism along with other risk factors. Material and Methods: A case-control study comprising of mothers of DS with and without CHD along with controls were recruited from a tertiary care center since 2018–2019. Genomic DNA was isolated followed by PCR-RFLP. Results: Mothers with age ≥35 years and having history of miscarriages have a higher risk of giving birth to DS with CHD (n = 35% and 42%, respectively). Mothers who carried the MTHFR 677CT/TT and MTRR 524CT/TT genotypes combination in the folic acid nonusers group during pregnancies had six-fold (OR = 6.909, *p*-value = 0.027; 95% CI—1.23 ± 38.51) and four-fold (OR = 4.75, *p*-value = 0.040; 95% CI—1.067 ± 21.44) increased odds of having a DS child with CHD, respectively, as compared to folic acid users. Conclusion: Maternal age, folic acid supplementation, and previous history of miscarriages is involved in the etiology of CHD in DS fetus in Indian population. Maternal MTHFR and MTRR polymorphisms are also involved in the occurrence of CHD and DS in Indian population when controlling for periconceptional folic acid supplementation. Limitations: Single-Centered Study

## 1. Introduction

Down syndrome (DS), also called as trisomy 21, is caused due to maternal non-disjunction of chromosome 21. The prevalence of DS is approximately 1–2 per 1000 live births, and it is characterized by the presence of mental retardation along with congenital anomalies, including congenital heart defects (CHD), Hirsprung’s disease, Alzheimer’s disease, leukemia, etc. CHD in DS account for 40 to 63.5% increased incidence of atrioventricular septal defects and ventricular defects in these population [1,2,3,4].

The maternal age and previous history of miscarriages are well-defined risk factors for DS. Additionally, few reports have showed the association of maternal age and previous history of miscarriages with CHD as well, respectively [5,6,7,8,9,10]. Wu et al., in 2013, conducted a survey to screen the effect of maternal age in association with the risk of birth of DS babies in England and have concluded that those women greater than the age of 35 years are at 20% higher risk of giving birth to DS babies [5]. The same finding was reported in a study conducted in 2016 and 2018, respectively, where the authors reported that advanced maternal age is one of the important risk factors for developing CHD in DS [6,7]. Feng et al. examined a relationship with maternal reproductive history and risk of developing CHD in DS. The authors identified previous history of miscarriages as one of the potential risk factors for developing CHD. However, its association in combination with CHD and DS remains unexplored and speculative in the Indian population.

Studies have investigated that folate, an important vitamin, plays essential role in reducing the risk of developing of cardiovascular diseases in during fetal life [11]. Folate pathway is governed by 5,10-methylenetetrahydrofolate reductase (*MTHFR*) and 5-methyltetrahydrofolate-homocysteine methyltransferase reductase (MTRR) enzymes encoded by MTHFR and MTRR gene. The polymorphism of MTRR and MTHFR in mothers have been previously linked to incidence of DS globally. Moreover, owing to the role of folate metabolism in cardiovascular development, the MTRR and MTHFR maternal polymorphisms may also pose a risk for CHD in DS offspring [11]. However, limited number of case-control studies have demonstrated MTHFR and MTRR polymorphisms as a maternal risk factors for CHD in DS, which provide conflicting and inconclusive results [11,12,13]. These inconclusive results can be due to differences in sample size and ethnicities of recruited study subjects.

Epidemiological studies have shown that folic acid supplementation can influence the occurrence of CHD and DS independently [11,12,13,14]. Coppedde et al. conducted a review shedding light on several published articles to explore the maternal risk factors in developing CHD, but most of them have inconclusive results [11]. A study conducted in 2017 also showed that folic acid supplementation before or during pregnancy reduced the risk of developing CHD [12]. Few studies or inconclusive results were reported from the studies conducted in the past that urge reviewing these factors, such as effect of maternal folic acid supplementation, previous history of miscarriages, and maternal age, in association with the occurrence of CHS in DS fetus in maternal subjects.

In the present study, we investigated the effect of genetic variants in maternal folate-metabolizing genes, such as MTHFR and MTRR, as a risk of CHD in DS fetus. In addition, the effect of maternal folic acid supplementation was inspected, which could influence folate metabolism. Additionally, we also examined other maternal risk factors, such as maternal age and previous history of miscarriages, for the occurrence of CHD in DS fetus.

## 2. Materials and Methods

### 2.1. Designs

This case-control study comprised of two groups of cases, including mothers of DS with CHD (Group I) and mothers of DS without CHD (Group II), along with age matched maternal controls who had previously delivered healthy offspring (Group III), and it was designed for analyzing the maternal risk factors for the occurrence of CHD in DS.

Settings: The study was conducted in a tertiary care and advanced research center in India from September 2018 till December 2019.

### 2.2. Participants

Group I, Group II, and Group III comprised of forty, sixty, and fifty mothers, respectively. Cases and control numbers were calculated using a formula on the basis of prevalence of DS (N = Z^2^PQ/d^2^, where z = statistic for the level of confidence, P = reported prevalence, Q = 100-P, and d = 5%–precision/error). The sample collection was obtained from those referred to the Department of Medical Genetics, Sanjay Gandhi Postgraduate Institute of Medical Sciences (SGPGIMS), Lucknow, and controls were healthy volunteers visiting general hospital, SGPGIMS, Lucknow, India. Inclusion criteria for cases were recruitment of those mothers who had delivered DS babies, and the study was further classified by the presence and absence of CHD along with DS in those offspring. All the clinical details of the study, including previous history of miscarriages, age, and folic acid supplementation, were recorded while interviewing and preparing the study questionnaire from the participant. Informed consent from participants was also collected at the same time. Other details, including mean maternal age and weight, total number of pregnancies, history and number of previous miscarriages, tobacco use, and educational level, were also recorded at the same time.

For studying the maternal age as a risk factor in the three above-mentioned study groups, mothers were sub-divided into two groups: one equal to and greater than 35 years and the other of less than 35 years of age.

#### Genotyping of MTHFR and MTRR Gene

Two milliliters of peripheral blood in ethylene diamine tetra acetic acid vial were collected from all cases and controls. Genomic DNA was isolated using standard phenol chloroform method followed by its quantification using Nanodrop Spectrophotometer (Thermo Scientific™ NanoDrop 2000, Waltham, MA, USA), and the DNA was stored 4 °C for further use. Polymerase chain reaction was used to amplify MTHFR and MTRR gene for both the variants (Table 1). The PCR-amplified products were digested using restrictions enzymes and were kept at 37 °C overnight, followed by the electrophoresis on a 3.5% agarose gel. (Table 1).

### 2.3. Risk Calculation and Statistical Analysis

The chi-square test was used to evaluate the genotypic frequencies and also to test for deviance from Hardy–Weinberg Equilibrium. The odds ratio (OR) was calculated to estimate the odds of CHD in DS individuals in maternal cases for various combinations of MTHFR and MTRR genotypes and maternal risk factors during pregnancy. The OR was also used determine the odds of having an offspring with CHD in DS in maternal MTHFR 677CT + TT versus the wild-type CC genotype and maternal MTRR 1298AC + CC versus the wild-type AA genotypes stratified by folic acid supplementation. For maternal age as a risk factor, subjects with age <35 or ≥35 years were recruited since increased risk of DS offspring among women over the age of 35 was previously reported. All analyses were performed using SPSS for Windows, version 16.0. The *p*-values were considered to be significant at 0.05, and 95% confidence intervals (95%CI) were reported.

## 3. Results

### 3.1. Maternal Risk Factors for the Occurrence of CHD in DS

The frequencies of maternal age ≥ 35 years in Group I, Group II, and Group III are 65%, 42%, and 40%, respectively. The frequency of maternal age < 35 years in Group I, Group II, and Group III are 35%, 58%, and 60%, respectively. The Table 2 shows that the mothers whose age is greater than 35 years have a higher risk of giving birth to DS babies with CHD (group I, N = 65%) compared to the other two groups (mothers of DS without CHD (N = 42%) and healthy controls mothers who have previously delivered normal and healthy babies (N = 40%)).

The frequency of folic acid supplementation was found to be highest in Group III (N = 90%), followed by Group II (N = 65%) and Group I (52%). In Group III, highest frequency was observed in participants taking folic acid supplementation (controls, 92%) compared to Group I (52%) and II (65%), which suggests that folic acid is associated with the occurrence of CHD in DS fetuses. (Table 2)

The frequency of mothers having previous history of any miscarriages shows that the highest risk of miscarriages influences the occurrence of CHD in DS fetuses in maternal subjects in Group I (N = 52%), and the least frequency was seen in Group III (N = 2%), respectively (Table 2).

### 3.2. Significance of Folic Acid Supplementation during Pregnancy

The risk of having CHD in DS offspring was analyzed by calculating the odds ratio in MTHFR and MTRR genotypes of Group I and Group II mothers. These two groups were distributed on the basis of intake of folic acid during pregnancy. Those mothers having genotypes MTHFR 677CT/TT along with folic acid supplementation during pregnancy had 1.909 odds ratio (*p*-value = 0.253; 95% CI = 0.628 ± 5.79), and the mothers who did not take folic acid during pregnancies had an increased odds ratio of 6.909 (*p*-value = 0.027; 95% CI—1.23 ± 38.51) of having a DS child with CHD (Table 3).

Similarly, mothers with genotype MTRR 524CT/TT along with folic acid supplementation during pregnancy had a odds ratio of 2.90 (*p*-value = 0.07; 95% CI:0.90 ± 9.29), and the those mothers who did not take folic acid during pregnancies had an increased odds ratio of 4.75 (*p*-value = 0.040; 95% CI—1.067 ± 21.44) of having a DS child with CHD. (Table 3).

## 4. Discussion

To date, limited data are available showing the association of the maternal risk factors (such as maternal age, previous history of miscarriages, and use of folic acid supplementation during pregnancy) and the occurrence of CHD in DS fetuses [11,12,13]. Therefore, in the present study, we studied MTHFR (C677T and A1298C) and MTRR (C524T and A66G) gene polymorphisms in Group I (mothers of DS with CHD), II (mothers of DS without CHD), and III (healthy age-matched mothers who had delivered healthy offspring) along with above-mentioned maternal risk factors.

Maternal age is a well-established risk factor for DS, and previous studies have concluded that women over 35 years of age have a higher probability of having an affected child with DS [12,15,16]. Along with these findings, in our study, we also observed an increased frequency of offspring with CHD in DS in mothers ≥ 35 years of age. These findings were in concordance with the findings reported in a recently published study in the south Indian population [12]. On the contrary, our findings were not in concordance with the study done by Chehab et al. in 2007, which reported that maternal age > 32 years was associated with a reduced occurrence of CHD in DS infants [12]. Previously, a study conducted by the National Down syndrome project reported that presence of ethnic differences influences the pattern of CHD in DS individuals. This might be the possible reason for our study’s different findings from the findings of Chehab et al., as a later study included subjects from the Lebanese population, while our subjects were from India [12]. Moreover, we used a more stringent age range (≥ 35 years) as compared to that selected by Chhab et al. (>32 years), which might cause differences in the findings.

Maternal folic acid supplementation during pregnancy is associated with the reduced the risk of CHD and DS, both independently [11,12,13,14]. Studies of maternal folic acid supplementation in the occurrence of CHD in DS suggest an involvement of folate pathway; yet, the association studies reported contradictory results. Since the folate pathway is governed by MTHFR (5,10-methylenetetrahydrofolate reductase) and MTRR (5-methyltetrahydrofolate-homocysteine methyltransferase reductase) enzymes encoded by MTHFR and MTRR gene, it can hence be concluded that the polymorphisms present in MTHFR and MTRR genes may alter the enzymatic activities. Thus, of the resulting gene product can be associated with the development of various malformations, including of CHD in DS [11]. Furthermore, various epidemiologic studies have shown that folic acid supplementation during pregnancy reduces the risk of CHD among offspring in DS [17,18,19]. In our study, we compared the association of mothers of DS with/without CHD who took folic acid supplementation and MTHFR and MTRR genotypes; and further, the study was stratified by their use of folic acid during pregnancy. Our results showed that mothers who carried the MTHFR 677CT or TT and MTRR 524CT or TT genotypes combination in the absence of folic acid supplementation during pregnancies had six-fold (OR = 6.909, *p*-value = 0.027; 95% CI—1.23 ± 38.51) and four-fold (OR = 4.75, *p*-value = 0.040; 95% CI—1.067 ± 21.44) increased odds of having a DS child with CHD, respectively, as compared to mothers who had taken the folic acid supplementation (Table 3). We found a positive association that existed between the maternal folic acid supplementation during pregnancy and risk of having DS offspring with CHD. A U.S.-based study conducted by Meijer et al. is the only available report in respect to maternal folic acid supplementation in the occurrence of CHD in DS fetuses. The results reported by Meijer et al. were in accordance with our study’s findings. [11].

The association between maternal history of miscarriage and risk of having a DS fetus in the future is well documented. In addition, previous studies have shown a positive association between maternal history of miscarriages and increased risk of heart diseases in DS fetuses (OR = 1.45; 95% CI = 1.18 to 1.78), whereas some studies have reported inconclusive results due to limited availability of data [7,19,20,21,22,23]. In line with these findings, interestingly, our study showed the increased incidence of developing CHD in DS fetuses in women having a history of miscarriages. The highest frequency of miscarriages was observed in mothers of DS with CHD (Group I), followed by mothers of DS without CHD (Group II) and then healthy control mothers (Group III) (Table 2).

## 5. Conclusions

Maternal risk factors, such as age, folic acid supplementation, and previous history of miscarriages, are involved as the potent maternal risk factors in the development of CHD in DS fetuses in the Indian population. Additionally, maternal MTHFR and MTRR genetic polymorphisms are also involved in the occurrence of CHD and DS in the Indian population along with folic acid supplementation during pregnancy. These findings can in turn be used as a maternal biomarker for the prevention of CHD in DS fetuses, leading to better diagnosis treatment and management of disease, which could open an window for reproductive counseling both before and during pregnancy.

## Figures and Tables

**Table 1:** PCR Conditions for Methylenetetrahydrofolate Reductase and Methionine Synthase Reductase Gene Polymorphisms.

Gene (Variants)	Primers	Annealing Temperature (T_a_ °C)	Amplicon Size (bp)	Restrictions Enzymes
MTHFR (C677T)	F-5′-CCTTGAACAGGTGGAGGCCAG-3′ R-5′-GCGGTGAGAGTGGGGTGGAG-3′.	65	196	Hinf I
MTHFR (A1298C)	F-5′-CTTTGGGGAGCTGAAGGACTACTA-3′ R-5′-CACTTTGTGACCATTCCGGTTTG-3′	62	163	Mbo II
MTRR (C524T)	F-5′-GTCAAGCAGAGGACAAGAG-3′ R-5′-AGAGACTCCTGCAGATGTAC-3′	60	247	Xho I
MTRR (A66G)	F-5′-GCAAAGGCCATCGCAGAAGACAT-3′ R-5′-AAACGGTAAACGGTAAAATCCACTGT-3′	60	126	Nde I

Abbreviations: MTHFR, methylenetetrahydrofolate reductase; MTRR, methionine synthase reductase.

**Table 2:** Maternal environmental risk factors for the occurrence of congenital heart defects in Down syndrome.

Maternal Risk	Group I (N = 40) n (%)	Group II (N = 60) n (%)	Group III (N = 50) n (%)
Maternal Age			
≥35	26 (65)	25 (42)	20 (40)
<35	14 (35)	35 (58)	30 (60)
Folic Acid supplementation	21 (52)	39 (65)	45 (90)
Miscarriages	21 (52)	5 (8.3)	1 (2)

Abbreviations: N, total number of participants; n (%), frequency in percentage; Group I, mothers of DS with CHD; Group II, mothers of DS without CHD; Group III, healthy control mothers.

**Table 3:** Genotypes in mothers with ds fetuses with chd versus ds fetuses without chd stratified by periconceptional folic acid use.

Folic Acid Users vs. Non-Users	with CHD	without CHD	OR (*p*-Value, 95% CI)
**USERS**			
MTHFR CC	12	28	1.909 (0.2538, 0.628 ± 5.79)
MTHFR TT + CT	09	11	
**NON-USERS**			
MTHFR CC	11	19	6.909 (0.027, 1.23 ± 38.51)
MTHFR TT + CT	08	02	
**USERS**			
MTHFR AA	15	33	2.20
MTHFR AC + CC	06	06	0.22 (0.608 ± 7.95)
**NON-USERS**			
MTHFR AA	10	14	3.08
MTHFR AC + CC	11	05	(0.098, 0.812 ± 11.67)
**USERS**			
MTRR CC	12	31	2.90 (0.07, 0.90 ± 9.29)
MTRR CT + TT	09	08	
**NON-USERS**			
MTRR CC	12	19	4.75 (0.040, 1.067 ± 21.44)
MTRR CT + TT	09	03	
**USERS**			
MTRR AA	14	26	1.0 (1.0, 0.32 ± 3.08)
MTRR AG + GG	07	13	
**NON-USERS**			
MTRR AA	14	18	9 (0.05, 0.988 ± 81.93)
MTRR AG + GG	07	01	

Abbreviations: MTHFR, methylenetetrahydrofolate reductase; MTRR, methionine synthase reductase; CHD, congenital heart defects; *p*-value > 0.005; OR, odds ratio.

## Data Availability

Not applicable.
